# Dynamic assessment of the inflammatory response in military personnel: a pilot study on ΔNLR and composite markers in operational environments

**DOI:** 10.25122/jml-2025-0056

**Published:** 2025-03

**Authors:** Florina-Diana Mihai, Emil-Tiberius Trasca, Dumitru Radulescu, Patricia-Mihaela Radulescu, Razvan Mercut, Elena-Irina Caluianu, Eleonora Daniela Ciupeanu-Calugaru, Dan Marian Calafeteanu, Georgiana-Andreea Marinescu, Suzana Danoiu

**Affiliations:** 1 UMF Craiova Doctoral School, University of Medicine and Pharmacy of Craiova, Craiova, Romania; 2 Department of Surgery, University of Medicine and Pharmacy of Craiova, Craiova, Romania; 3 Dr. Stefan Odobleja Military Emergency Clinical Hospital, Craiova, Romania; 4 Department of Pneumology, University of Medicine and Pharmacy of Craiova, Craiova, Romania; 5 Department of Plastic and Reconstructive Surgery, University of Medicine and Pharmacy of Craiova, Craiova, Romania; 6 Department of Biology and Environmental Engineering, University of Craiova, Craiova, Romania; 7 Department of Orthopedics, University of Medicine and Pharmacy of Craiova, Craiova, Romania; 8 Department of Pathophysiology, University of Medicine and Pharmacy of Craiova, Craiova, Romania

**Keywords:** NLR, ΔNLR, SIRI, SII, IIC, systemic inflammation, operational stress, military

## Abstract

In this pilot study, we investigated immune alterations in 178 military personnel exposed to extreme operational stress. We focused on the neutrophil-to-lymphocyte ratio (NLR) and its change (ΔNLR) alongside composite inflammatory indices— Systemic Inflammation Response Index (SIRI), Systemic Immune-Inflammation Index (SII), and Inflammatory Index Cumulative (IIC). Blood analyses performed before and after deployment revealed a significant post-mission increase in NLR (1.9671±±±0.9174 vs. 1.6079±±±0.4973 pre-deployment), yielding an average ΔNLR of 0.3592±±±0.7642 (*P* < 0.0001). While basophil counts and several biochemical markers remained stable, notable changes in neutrophils and composite indices suggest a complex inflammatory activation. Importantly, correlation analyses confirmed that despite marked shifts in absolute values, the relative relationships between pre- and post-deployment measurements (e.g., NLR: r = 0.5533, *P* < 0.0001) were maintained. These findings imply that ΔNLR, together with SIRI, SII, and IIC, may serve as valuable biomarkers for dynamically monitoring the inflammatory response in military contexts, thereby enabling early identification of individuals at increased inflammatory risk.

## INTRODUCTION

The health challenges faced by military personnel exposed to extreme operational conditions have emerged as a critical issue in modern military medicine [1]. In addition to physical stressors such as intense exertion, variable climatic conditions, and sleep deprivation, these individuals encounter psychological and biological stress that can trigger systemic inflammation and oxidative stress [2,3]. Prolonged inflammatory responses have been associated with increased morbidity and mortality, emphasizing the need for early detection of inflammatory biomarkers to guide preventive and therapeutic strategies [4]. Furthermore, understanding these responses is essential for designing targeted interventions to reduce health risks in high-stress military settings.

Recent evidence has underscored the neutrophil-to-lymphocyte ratio (NLR) as a sensitive marker of both inflammation and oxidative stress, with established links to cardiovascular risk and other pathologies [5]. Moreover, the variation in NLR between pre- and post-deployment measurements (ΔNLR) appears to reflect acute immune modifications in response to high-stress scenarios [6]. In parallel, composite indices such as the Systemic Inflammation Response Index (SIRI), Systemic Immune-Inflammation Index (SII), and Inflammatory Index Cumulative (IIC) offer a more integrated view of the inflammatory process by combining multiple hematological parameters [7,8]. These composite indices are particularly valuable because they integrate diverse aspects of the immune response, providing a more complete view of inflammation than single biomarkers alone. Evaluating these biomarkers in a military setting could substantially enhance risk stratification and health monitoring in operational environments [9]. Therefore, the primary aim of this study was to assess ΔNLR as a marker of inflammatory response in military personnel before and after deployment while also exploring its relationship with composite inflammatory indices and potential demographic or metabolic influences [10]. This approach facilitates the detection of acute inflammatory changes and supports the development of user-friendly monitoring tools that can be implemented in field conditions. Our findings could help establish a user-friendly monitoring approach for early detection of soldiers at high risk for complications, such as infections, cardiovascular events, or prolonged fatigue [1,11].

## MATERIAL AND METHODS

### Study design and population

This retrospective investigation was conducted at Dr. Stefan Odobleja Military Hospital in Craiova, Romania, between October 1, 2020, and October 31, 2024. Of the 236 military personnel initially screened, 58 were excluded based on predefined criteria, resulting in a final cohort of 178 participants.

### Inclusion and exclusion criteria

**Figure 1:**
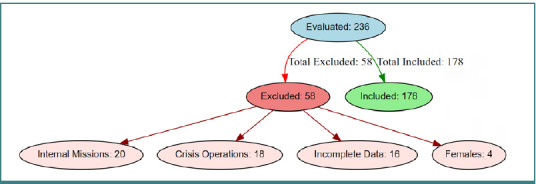
Study population selection flowchart for military personnel

Only complete clinical and laboratory records of soldiers deployed on international missions—whether for national defense within NATO alliances, peacekeeping, or counter-terrorism operations—were included (Figure 1).

Data were retrieved from the hospital’s electronic system. Exclusion criteria encompassed:

- Personnel involved exclusively in domestic missions (given the focus on external operational stress).
- Subjects engaged in crises (e.g., emergency interventions, international tensions) where biological and psychological parameters might be substantially altered.
- Incomplete clinical records.
- Female subjects, due to their very limited representation (*n* = 4), which might introduce statistical bias.

### Statistical analysis

Data were processed using SPSS version 26.0. Descriptive statistics (mean, standard deviation, median, percentiles, minimum, and maximum) were calculated for all variables. A paired *t-*test compared pre- and post-deployment values. Pearson’s correlation coefficients were used to quantify associations among key inflammatory markers (NLR, SIRI, SII, IIC). Statistical significance was set at *P* < 0.05.

## RESULTS

**Figure 2:**
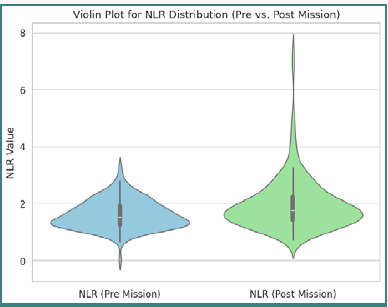
Violin plot of NLR distribution before and after the mission

The analysis of 178 military personnel revealed significant immunological changes post-deployment. Mean NLR values increased from 1.6079±±±0.4973 before deployment to 1.9671±±±0.9174 afterward, corresponding to an average ΔNLR of 0.3592±±±0.7642 (Figure 2).

A paired *t-*test confirmed the significance of this increase (t = 6.2712, *P* < 0.0001) (Table 1).

**Table 1:** Descriptive statistics for NLR

Statistic	Pre-deployment	Post-deployment	Difference (ΔNLR)
Count	178	178	178
Mean	1.6079 ± 0.4973	1.9671 ± 0.9174	0.3592 ± 0.7642
Minimum	0.0232	0.7373	–0.7388
25^th^ Percentile	1.2352	1.4196	–0.0322
Median	1.5263	1.7609	0.2324
75^th^ Percentile	1.9086	2.2266	0.5063
Maximum	3.2605	7.2868	5.6081

This indicates that, on average, the inflammatory activity was significantly elevated post-deployment. The broad range of ΔNLR (–0.7388 to 5.6081) also denotes substantial interindividual differences.

A more detailed comparison of pre- vs. post-deployment biomarkers revealed stability in some parameters (e.g., basophils, cholesterol) alongside substantial changes in others. For instance, basophil counts remained essentially the same (0.0300 ± 0.0210 vs. 0.0302 ± 0.0179, *P* = 0.8706), whereas creatinine, glucose, and other metabolic indices showed no marked differences. By contrast, neutrophil counts increased significantly from 3.5667 ± 1.1780 to 4.3315 ± 1.8717 (*P* < 0.0001). SIRI, SII, and IIC also increased notably (Table 2).

**Table 2:** Descriptive statistics for biological markers

Parameter	Pre-deployment (Mean ± SD)	Post-deployment (Mean ± SD)	*P* value
Basophils	0.0300 ± 0.0210	0.0302 ± 0.0179	0.8706
Cholesterol	191.8365 ± 38.2113	193.5056 ± 36.8292	0.6750
Creatinine	0.9195 ± 0.2360	0.9096 ± 0.1575	0.6405
Eosinophils	0.4430 ± 3.4439	0.1969 ± 0.1347	0.3420
Glucose	96.1292 ± 9.5779	96.7253 ± 8.3768	0.5324
Neutrophils	3.5667 ± 1.1780	4.3315 ± 1.8717	<0.0001
Hematocrit	44.3700 ± 4.1755	43.9360 ± 2.5909	0.2399
Hemoglobin	15.2860 ± 2.1293	15.3404 ± 1.0019	0.7576
Lymphocytes	2.7494 ± 6.3273	2.2892 ± 0.6303	0.3355
Leukocytes (WBC)	29.7516 ± 2.7240	30.0820 ± 1.5715	0.1621
Chem	34.1843 ± 3.4068	34.9157 ± 0.9489	0.0063
Volumetric Reticulocytes	87.6629 ± 10.2050	86.1770 ± 4.1087	0.0728
Monocytes	2.3290 ± 23.2673	0.6390 ± 0.1791	0.3339
VTM	10.7828 ± 1.2705	10.5983 ± 0.9544	0.1223
Plateletcrit	0.3197 ± 0.9571	0.2425 ± 0.0472	0.2840
Platelets	232.6348 ± 58.4372	231.4213 ± 52.5230	0.8369
Erythrocytes	5.3253 ± 3.2619	5.1099 ± 0.3860	0.3829
RDW	13.3882 ± 5.4659	13.0697 ± 0.8705	0.4436
GOT	24.9213 ± 7.7914	22.7303 ± 7.5626	0.0074
GPT	29.8041 ± 13.2865	27.1629 ± 13.9207	0.0679
Triglycerides	118.4831 ± 55.0579	138.5730 ± 83.6731	0.0079
ESR (VSH)	8.0843 ± 5.1769	7.2303 ± 5.3718	0.1276
Leukocyte count (Chem)	6.6485 ± 1.7057	7.4869 ± 2.3058	0.0001
NLR	1.6079 ± 0.4973	1.9671 ± 0.9174	<0.0001
PLR	106.9892 ± 31.1618	107.3743 ± 34.8948	0.9126
MLR	0.2814 ± 0.2604	0.2885 ± 0.0796	0.7285
SIRI	0.9853 ± 0.6464	1.2892 ± 0.8054	0.0001
SII	379.0032 ± 163.7715	463.9125 ± 272.3108	0.0004
IIC	1.8352 ± 0.5923	2.2240 ± 1.1023	<0.0001

These data demonstrate that while certain parameters (e.g., basophils, cholesterol, creatinine) did not show significant changes, others, particularly neutrophils, leukocyte count (Chem), SIRI, SII, and IIC, exhibited considerable alterations indicative of an active inflammatory response.

**Figure 3:**
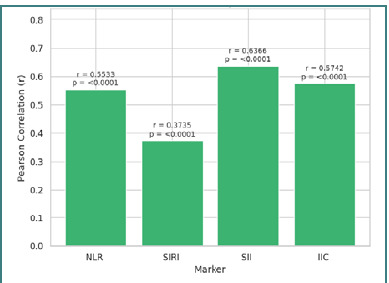
Pearson correlation coefficients for inflammatory markers

Pearson correlation analysis evaluated the consistency of immunological changes pre- and post-deployment. A moderate-to-strong correlation emerged for NLR (r = 0.5533, *P* < 0.0001), and significant correlations were noted for SIRI (r = 0.3735, *P* < 0.0001), SII (r = 0.6366, *P* < 0.0001), and IIC (r = 0.5742, *P* < 0.0001) (Figure 3).

**Figure 4:**
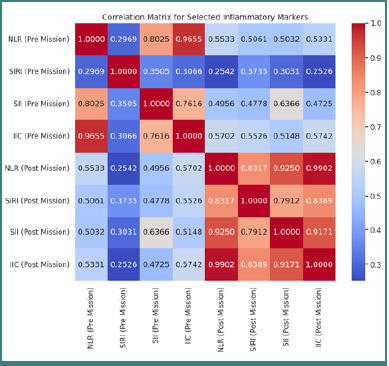
Correlation matrix of pre-mission and post-mission markers

These results indicate that although the absolute values changed significantly post-deployment, the relative relationships between the pre- and post-deployment measurements remained consistent. Furthermore, a correlation matrix demonstrated strong interrelations among the pre-deployment markers and post-deployment markers, thereby confirming the reliability of these indicators for dynamic monitoring of the inflammatory response (Figure 4).

**Figure 5:**
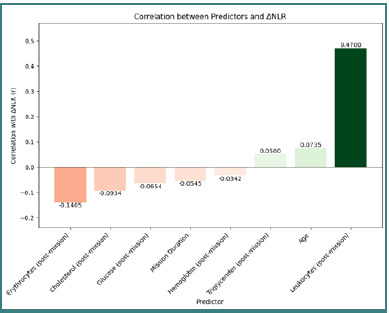
Correlation between predictors and ΔNLR

An additional correlation matrix (Figure 5) was employed to evaluate the relationships between the studied variables and ΔNLR, highlighting the following associations: age (r = 0.0735), mission duration (r = –0.0545), cholesterol (r = –0.0934), glucose (r = –0.0654), post-deployment leukocyte count (Chem: r = 0.4700), triglycerides (r = 0.0500), hemoglobin (r = –0.0342), and post-deployment erythrocyte count (r = –0.1405).

**Figure 6:**
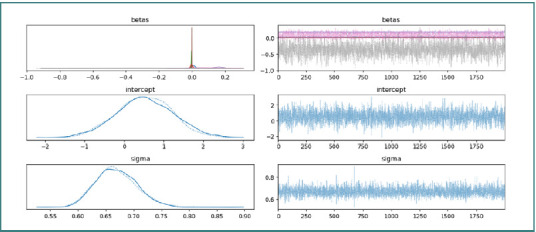
Trace plot of MCMC chains for the Bayesian model

These findings indicate a strong positive association between post-deployment leukocyte count and ΔNLR, suggesting that increased leukocytes were linked to an intensified inflammatory response, whereas an elevated post-deployment erythrocyte count was associated with a decrease in ΔNLR, possibly reflecting a protective mechanism. To assess the individual effects of predictors on ΔNLR, a Bayesian analysis was performed (Figure 6).

The model generated robust estimates, yielding a mean coefficient for post-deployment leukocytes of +0.163 (sd = 0.022, 95% credible interval: [0.117, 0.205]), indicating a predictable increase in ΔNLR with higher leukocyte counts. In contrast, post-deployment erythrocytes had a mean coefficient of –0.361 (sd = 0.177, 95% credible interval: [–0.703, –0.016]), underscoring their counterbalancing effect on the inflammatory response. The convergence of the MCMC chains, as demonstrated by the trace plot, confirms the stability of these estimates.

**Figure 7:**
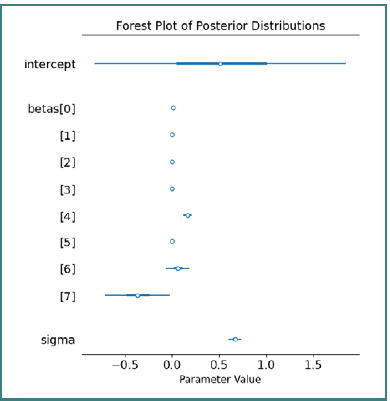
Forest plot of credible intervals for model parameters

A forest plot visually summarizes the credible intervals for all model parameters, clearly delineating predictors with major influence from those with a reduced effect on ΔNLR (Figure 7).

**Figure 8:**
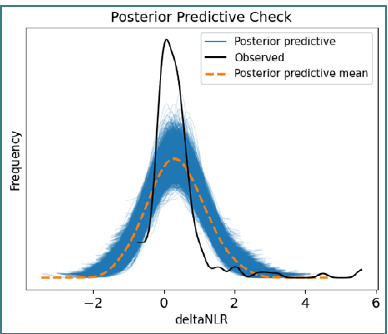
Posterior predictive check of ΔNLR distribution

This plot facilitates a rapid evaluation of the statistical significance of each coefficient, aiding in the interpretation of the impact of the studied variables. The model’s predictive performance was further assessed via a posterior predictive check, which revealed a mean ΔNLR of 0.3572, a standard deviation of 0.7944, and a wide credible interval ranging from –1.1570 to 1.9705 (Figure 8).

These results underscore the high variability of the inflammatory response among subjects and the model’s capacity to reproduce the observed data distribution.

**Figure 9:**
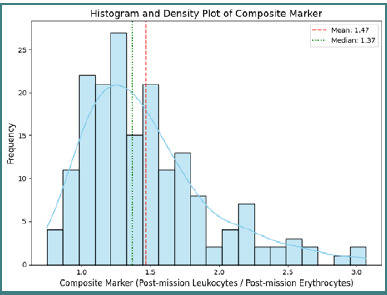
Distribution of the composite marker (post-deployment leukocytes/erythrocytes)

Analysis of the composite marker—defined as the ratio of post-deployment leukocytes to erythrocytes—in 178 observations yielded a mean value of 1.4708, a standard deviation of 0.4571, extreme values between 0.7496 and 3.0642, a 25^th^ percentile of 1.1310, a median of 1.3718, and a 75^th^ percentile of 1.6850 (Figure 9).

**Figure 10:**
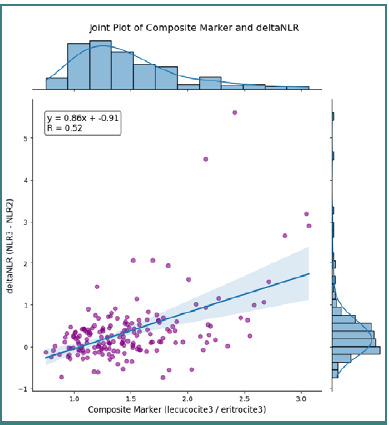
A joint plot of the composite marker and ΔNLR

The relationship between the composite marker and ΔNLR was further explored using a joint plot, demonstrating that higher composite marker values were associated with increased ΔNLR (Figure 10).

**Figure 11:**
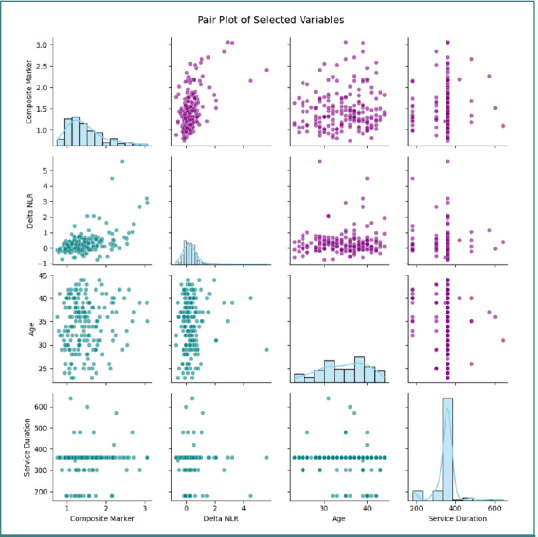
Pair plot: marginal distributions and bivariate relationships among the composite marker, ΔNLR, age, and mission duration

A clear trend was evident: subjects with a composite marker above 1.6850 (indicative of leukocyte predominance) exhibited higher ΔNLR values, suggesting a potential causal link between these variables. Finally, a pair plot incorporating the composite marker, ΔNLR, age, and mission duration provided an integrated view of the marginal distributions and bivariate relationships. The marginal plots depict the distribution shape of each variable (as indicated by the descriptive statistics), while the scatter plots illustrate the complex interactions among these parameters, thereby contributing to a deeper understanding of the dynamics of the inflammatory response (Figure 11).

In summary, the composite marker (i.e., the leukocyte-to-erythrocyte ratio) and ΔNLR appear to be a robust and innovative indicator for evaluating inflammation in military personnel. These parameters may be employed as stand-alone post-deployment markers and for dynamic monitoring throughout mission execution, offering new insights into understanding and managing the inflammatory response under extreme operational conditions.

## Discussion

The analysis of 178 military personnel revealed a substantial post-deployment increase in NLR, underscoring a strong immunologic response triggered by the rigors of operational stress [5,6,12]. These findings further support the potential for early intervention strategies to mitigate stress-induced complications. The observed increase in ΔNLR, correlated with elevated neutrophil counts, supports the hypothesis that physical and psychological stressors commonly encountered in operational theaters (e.g., intense exertion, sleep deprivation, high stress) may trigger systemic inflammation [13]. This observation is in line with previous studies showing that NLR reflects both systemic inflammation and oxidative stress induced by increased secretion of glucocorticoids and catecholamines [1,5,14].

In addition to NLR, composite markers such as SIRI, SII, and IIC are of particular interest. Consistent with other studies [7,8], our findings suggest that these indices offer a more comprehensive assessment of the inflammatory response by integrating data on neutrophils, lymphocytes, platelets, and, in some cases, other metabolic or hormonal parameters [15]. Our results underscore the importance of these composite indices in capturing the multifaceted nature of inflammation in high-stress environments, as supported by recent literature [16]. The significant post-deployment increases in SIRI, SII, and IIC may indicate a complex proinflammatory state that goes beyond the response to physical exertion, involving neuroendocrine and genetic mechanisms [2,10,17]. This integrated perspective suggests that employing a composite score incorporating ΔNLR with these indices could enhance risk stratification for immediate and long-term complications [18].

Although the literature suggests that factors such as age, mission duration, or cholesterol levels might influence the inflammatory response, no significant correlations were found in this study [2,14]. Possible explanations include the limited sample size, the homogeneity of the studied population, or the influence of unexamined factors (e.g., genetic predisposition, nutrition, medical history). It is noteworthy that some studies have reported significant associations in more heterogeneous groups, indicating that future research with larger and more diverse samples may reveal additional insights [3]. Other studies have highlighted that subgroups with dyslipidemia may exhibit a more pronounced inflammatory response; however, confirmation of this hypothesis would require a multicentric design and subgroup-focused analyses [4,10,17].

From an operational perspective, the findings suggest that pre- and post-deployment monitoring of ΔNLR and composite indices (SIRI, SII, IIC) could facilitate the early detection of military personnel predisposed to an excessive inflammatory response [1,6]. Such screening, which can be performed relatively simply via periodic blood tests, would enable personalized interventions—such as tailored training programs, stress reduction techniques, or nutritional counseling—to maintain operational performance and reduce morbidity [5,19]. Moreover, the implementation of digital monitoring tools could further streamline such efforts and support timely clinical decision-making [11]. The literature consistently underscores the importance of proactive inflammation management in preserving the response capacity and overall health of military personnel [2,9,13]. Our study's findings align with those reported in a previous investigation by our team, which demonstrated that incorporating ΔNLR into an integrated score (IIRPM) can provide a sensitive assessment of inflammatory risk among military personnel [20]. These results encourage further exploration into reliable, composite scoring systems for operational use.

However, this study has several limitations. First, the relatively small sample size and the absence of in-depth analyses of other biomarkers (e.g., IL-6, CRP) limit the generalizability of our findings [10]. Future research should incorporate a broader panel of biomarkers to validate and extend these preliminary observations. Second, psycho-emotional factors that might modulate the inflammatory response (e.g., anxiety, post-traumatic stress scores) were not evaluated. Integrating psychological assessments could provide a broader understanding of the interplay between mental stress and systemic inflammation. Third, since the sample was drawn from a single operational region, variations among military personnel from different regions or engaged in other types of missions cannot be ruled out [15,17]. Thus, multicentric studies with larger and more diverse cohorts are recommended for future investigations. Future large-scale, longitudinal studies incorporating cluster analyses and extended follow-up post-repatriation are warranted [7,21]. Such studies will be instrumental in refining our understanding of the dynamic interplay between operational stress and systemic inflammation.

## CONCLUSION

This study confirms that ΔNLR is a sensitive marker for the activation of the inflammatory response in military personnel, as evidenced by a significant post-deployment increase in NLR values. Furthermore, the composite indices (SIRI, SII, IIC) exhibited notable increases, suggesting the presence of a complex inflammatory process. These findings underscore the potential for integrating ΔNLR and composite markers into operational health monitoring protocols to optimize the early detection of stress-related complications. Integrating these biomarkers into a composite score may facilitate the rapid identification of individuals at high risk for post-deployment complications and enable the implementation of personalized interventions (e.g., stress management, nutritional adjustments, targeted inflammatory monitoring). Such composite scoring systems could pave the way for real-time monitoring and proactive care in military settings. Future studies with larger sample sizes and more comprehensive analyses—including additional psycho-emotional and metabolic factors and a multicentric approach—are recommended to validate and strengthen these conclusions. These further investigations will be essential in establishing the clinical utility and predictive accuracy of these biomarkers in diverse military populations.

## References

[1] Afari ME, Bhat T (2016). Neutrophil to lymphocyte ratio (NLR) and cardiovascular diseases: an update. Expert Rev Cardiovasc Ther.

[2] Carroll D (2006). Inflammation and oxidative damage during exam stress. Psychophysiology.

[3] Fink-Neuboeck N, Lindenmann J, Bajric S, Maier A, Riedl R, Weinberg AM (2016). Clinical impact of interleukin 6 as a predictive biomarker in the early diagnosis of postoperative systemic inflammatory response syndrome after major thoracic surgery: A prospective clinical trial. Surgery.

[4] Cho KI, Ann SH, Singh GB, Her SH, Shin ES (2016). OS 01-03 Neutrophil to lymphocyte ratio is closely related with blood pressure level in hypertensive individuals without cardiovascular diseases: data from the Korean registry of target organ damages (KorHR). J Hypertens.

[5] Wang S, Pan X, Jia B, Chen S (2023). Exploring the Correlation Between the Systemic Immune Inflammation Index (SII), Systemic Inflammatory Response Index (SIRI), and Type 2 Diabetic Retinopathy. Diabetes Metab Syndr Obes.

[6] Yun S, Yi HJ, Lee DH, Sung JH (2021). Systemic Inflammation Response Index and Systemic Immune-inflammation Index for Predicting the Prognosis of Patients with Aneurysmal Subarachnoid Hemorrhage. J Stroke Cerebrovasc Dis.

[7] Radulescu PM, Davitoiu DV, Baleanu VD, Padureanu V, Ramboiu DS, Surlin MV, Bratiloveanu TC, Georgescu EF, Streba CT, Mercut R, Caluianu EI, Trasca ET, Radulescu D (2022). Has COVID-19 Modified the Weight of Known Systemic Inflammation Indexes and the New Ones (MCVL and IIC) in the Assessment as Predictive Factors of Complications and Mortality in Acute Pancreatitis?. Diagnostics (Basel).

[8] Beckner ME, Main LC, Tait JL, Nindl BC (2021). Circulating biomarkers associated with performance and resilience during military operational stress. Eur J Sport Sci.

[9] Xia Y, Xia C, Wu L, Li Z, Li H, Zhang J (2023). Systemic Immune Inflammation Index (SII), System Inflammation Response Index (SIRI) and Risk of All-Cause Mortality and Cardiovascular Mortality: A 20-Year Follow-Up Cohort Study of 42,875 US Adults. J Clin Med.

[10] Bartlett EK, Flynn JR, Panageas KS, Ferraro RA, Sta Cruz JM, Postow MA, Coit DG, Ariyan CE (2020). High neutrophil-to-lymphocyte ratio (NLR) is associated with treatment failure and death in patients who have melanoma treated with PD-1 inhibitor monotherapy. Cancer.

[11] Maculewicz E, Pabin A, Kowalczuk K, Dziuda Ł, Białek A (2022). Endogenous Risk Factors of Cardiovascular Diseases (CVDs) in Military Professionals with a Special Emphasis on Military Pilots. J Clin Med.

[12] Chen Z, Li W, Tang Y, Zhou P, He Q, Deng Z (2024). The neutrophil-lymphocyte ratio predicts all-cause and cardiovascular mortality among United States adults with COPD: results from NHANES 1999-2018. Front Med (Lausanne).

[13] Biyik M, Biyik Z, Asil M, Keskin M (2022). Systemic Inflammation Response Index and Systemic Immune Inflammation Index Are Associated with Clinical Outcomes in Patients with Acute Pancreatitis?. J Invest Surg.

[14] Mustafa M, Daoub A (2022). The neutrophil-lymphocyte ratio (NLR) as a predictor of coronary artery disease severity. Cardiol Res Pract.

[15] Mihai F-D, Trasca E-T, Radulescu P-M, Mercut R, Caluianu E-I, Ciupeanu-Calugaru ED (2025). Advanced Assessment of Oxidative Stress and Inflammation in Military Personnel: Development of a Novel IIRPM Score Using Artificial Intelligence. Diagnostics (Basel).

[16] O'Connor D, Griffin ML, O'Sullivan JS, Millar S, O'Keeffe J, Bird BR, Deady S, Murphy CG (2015). Abstract P6-08-35: Pre-surgical neutrophil-to-lymphocyte ratio (NLR) is a prognostic indicator of recurrence free and overall survival in breast cancer patients undergoing primary surgery. Cancer Res.

[17] Beckner ME, Main L, Tait JL, Martin BJ, Conkright WR, Nindl BC (2022). Circulating biomarkers associated with performance and resilience during military operational stress. Eur J Sport Sci.

[18] Kargl CK, Gage CR, Forse JN, Koltun KJ, Bird MB, Lovalekar M (2024). Inflammatory and Oxidant Responses to Arduous Military Training: Associations with Stress, Sleep, and Performance. Med Sci Sports Exerc.

[19] Avey S, Chatterjee M, Manyakov NV, Cooper P, Sabins N, Mosca K (2024). Using a wearable patch to develop a digital monitoring biomarker of inflammation in response to LPS challenge. Clin Transl Sci.

[20] Makras P, Koukoulis GN, Bourikas G, Papatheodorou G, Bedevis K, Menounos P (2005). Effect of 4 weeks of basic military training on peripheral blood leucocytes and urinary excretion of catecholamines and cortisol. J Sports Sci.

[21] Bennett N, Lawrence-Wood E, McFarlane A (2024). Is inflammatory change associated with psychological risk and resilience in high-risk military personnel?. BMJ Mil Health.

